# Construction of a DDR-related signature for predicting of prognosis in metastatic colorectal carcinoma

**DOI:** 10.3389/fonc.2023.1043160

**Published:** 2023-02-01

**Authors:** Maohua Wei, Junyan Su, Jiali Zhang, Siyao Liu, Jia Ma, Xiang peng Meng

**Affiliations:** ^1^ Department of General Surgery, Dalian Medical University, Dalian, China; ^2^ Department of Scientific Research Projects, ChosenMed Technology Co. Ltd., Beijing, China; ^3^ Department of Gastroenterology, The Fourth Affiliated Hospital of China Medical University, Shenyang, China; ^4^ Department of General Surgery, Shengjing Hospital of China Medical University, Shenyang, China

**Keywords:** metastatic colorectal cancer, prognostic, DDRscore, therapy response, predictor

## Abstract

**Background:**

Colorectal cancer (CRC) is the third most prevalent malignancy and the one of most lethal cancer. Metastatic CRC (mCRC) is the third most common cause of cancer deaths worldwide. DNA damage response (DDR) genes are closely associated with the tumorigenesis and development of CRC. In this study, we aimed to construct a DDR-related gene signature for predicting the prognosis of mCRC patients.

**Methods:**

The gene expression and corresponding clinical information data of CRC/mCRC patients were obtained from Gene Expression Omnibus (GEO) and The Cancer Genome Atlas (TCGA) databases. A prognostic model was obtained and termed DDRScore by the multivariate Cox proportional hazards regression in the patients with mCRC. The Kaplan-Meier (K-M) and Receiver Operating Characteristic (ROC) curves were employed to validate the predictive ability of the prognostic model. Gene Ontology (GO) and Kyoto Encyclopedia of Genes and Genomes (KEGG) pathway were performed for patients between the high-DDRscore and low-DDRscore groups.

**Results:**

We constructed a prognostic model consisting of four DDR-related genes (EME2, MSH4, MLH3, and SPO11). Survival analysis showed that patients in the high-DDRscore group had a significantly worse OS than those in the low-DDRscore group. The area under the curve (AUC) value of the ROC curve of the predictive model is 0.763 in the training cohort GSE72970, 0.659 in the stage III/IV colorectal cancer (CRC) patients from The Cancer Genome Atlas (TCGA) data portal, and 0.639 in another validation cohort GSE39582, respectively. GSEA functional analysis revealed that the most significantly enriched pathways focused on nucleotide excision repair, base excision repair, homologous recombination, cytokine receptor interaction, chemokine signal pathway, cell adhesion molecules cams, ECM-receptor interaction, and focal adhesion.

**Conclusion:**

The DDRscore was identified as an independent prognostic and therapy response predictor, and the DDR-related genes may be potential diagnosis or prognosis biomarkers for mCRC patients.

## 1 Introduction

Colorectal cancer (CRC) is the third most prevalent malignancy and the second most lethal cancer, with an anticipated 1.9 million diagnoses and 0.9 million deaths globally in 2020 (1). The incidence of colon cancer has increased significantly, with most patients in the moderate and advanced stages at the time of diagnosis. According to the Chinese Guidelines for the Diagnosis and Treatment of CRC (2020 Edition) issued by the National Health Commission of China, the incidence and mortality of CRC in China ranks the third and fifth among all malignant tumors, with 376,000 new cases and 1.91 million deaths, it is much higher in urban areas than in rural areas.

Metastasis is the leading cause of cancer-related mortality. Metastatic Colorectal cancer (mCRC) is the third leading cause of cancer death globally, and the incidence of CRC, particularly mCRC, is increasing among younger people (2, 3). Patients with localized CRC have a 5-year survival rate of 85% to 90%, while the mCRC patients were just about 12-14% (4, 5). Finding reliable prognostic indicators to select high-DDRscore mCRC patients is critical for improving survival rates.

DNA damage response (DDR) is a cell-autonomous response to DNA damage, both endogenous and external. The DDR signaling pathway consists of eight branch pathways, namely mismatch repair (MMR), base excision repair (BER), nucleotide excision repair (NER), homologous recombination repair (HRR), nonhomologous end-joining (NHEJ), checkpoint factors (CPF), Fanconi anemia (FA), and translesion DNA synthesis (TLS). These DDR pathways mainly existed to perform the function of genome maintenance, thus preserving the genomic integrity (6, 7). Germline alterations in essential DDR genes cause tumor susceptibility, and various cancers carry somatic mutations that impair DDR (8). Recent studies suggest that a subset of CRCs is characterized by DDR genes’ germline and/or somatic genetic defects (9–12). Germline pathogenic variants of BRCA1 are rising as a risk factor for CRC, as BRCA1/2 alterations have been associated with early-onset CRC (13, 14). The prevalence of somatic DDR defects in CRC ranges between 10% and 30% (14, 15).

With only a few fragmented pieces of evidence on their clinical impact accessible, the role of DDR in mCRC is still largely unclear (16, 17). In this study, we identified the DDR genes in public datasets and constructed a prognostic model using multivariate Cox regression analysis to explore the potential role of DDR genes and accurately predict the prognosis of mCRC.

## 2 Material and method

### 2.1 Datasets and patients

We download three publicly available datasets of CRC from the Gene Expression Omnibus (GEO) (https://www.ncbi.nlm.nih.gov/geo/) and TCGA-COAD (https://xenabrowser.net/datapages). GSE72970 dataset as training corhot contains 21655 gene expressions of the 124 patients with mCRC. We download the gene expression data for stage III/IV colorectal cancer (CRC) patients from the Genome Data Commons (GDC) data portal and the GSE39582 dataset from the same Platform Affymetrix U133 Plus 2.0, a total of 392 CRC patients with 21665 gene expression as validation cohorts. The clinical characteristics of patients in these three cohorts are summarized in **Table 1**. From a previous study reported by Wang et.al (18)., we collected 233 DDR-related genes of 8 pathways. Tumor mutational burden (TMB) was determined by analyzing the number of somatic mutations per megabase. The TMB value≥10 mut/Mb was defined as the cutoff value of high TMB (TMB-H), and the TMB value<10 mut/Mb was determined as low TMB (TMB-L). We defined the overall survival (OS) of 2-years as a cutoff value of prognosis group.

**Table 1:** Characteristics of the colorectal carcinoma patients from training and validate datasets (GSE72970、GES39582 and TCGA-COAD).

Clinical characteristics	GSE72970			GSE39582			TCGA		
	High-DDRscore (N=62)	Low-DDRscore (N=62)	*P*-value	High-DDRscore (N=53)	Low-DDRscore (N=339)	*P*-value	High-DDRscore (N=25)	Low-DDRscore (N=125)	*P*-value
Age									
>65	29	21	0.200	34	198	0.522	11	57	1.000
<=65	33	41		19	141		14	68	
Gender									
male	37	37	1.000	28	181	1.000	13	66	1.000
female	25	25		25	158		12	59	
pT/T									
pT1/T1	1	–	0.541	1	12	0.266	0	1	0.000
pT2/T2	4	3		8	25		0	8	
pT3/T3	24	26		32	228		13	100	
pT4/T4	18	29		12	74		12	16	
pTX/TX	15	14		0	0		0	0	
pN/N									
pN0/N0	8	6	0.611	32	181	0.700	4	2	0.002
pN1/N1	16	12		13	91		11	79	
pN2/N2	23	30		7	63		10	44	
pNX/N3	15	14		1	4		0	0	
M									
M0	–	–		48	315	0.744	12	75	0.502
M1	–	–		5	24		9	32	
MX	–	–		0	0		4	18	
Stage									
I-II	–	–		32	179	0.368	–	–	0.413
III	–	–		16	136		16	93	
IV	–	–		5	24		9	32	
tumor location									
left	23	27	0.729	–	–		–	–	
right	17	14		–	–		–	–	
other	22	21		–	–		–	–	
metastase									
yes	62	62	NA	21	160	0.379	25	125	NA
no	0	0		32	179		0	0	
Status									
Alive	7	25	0.000	34	245	0.293	18	106	0.413
Dead	55	37		19	94		7	19	
OS(months)									
mOS	17.6(2.3-61.9)	31.4(3.4-76.1)		46(2-132)	55(1-201)		16.6(4.8-56.2)	24.9(0.2-135.6)	
PFS(months)									
mPFS	7.2(1.4-61.9)	11.6(1.5-76.1)		–	–		–	–	
RFS(months)									
mRFS	–	–			–		–	–	
Therapy									
Chemotherapy	53	42	0.034	53	339	NA	–	–	
Chemotherapy+Targeted therapy	9	20		0	0		–	–	
Response status									
CR	2	6	0.030	–	–		–	–	
PR	25	30		–	–		–	–	
SD	20	22		–	–		–	–	
PD	15	4		–	–		–	–	

pT/T, pathology Tumor/Tumor; pN/N, pathology Node/Node; M, metastasis; OS, Overall survival; mOS, median overall survival; PFS, progression-free survival; mPFS, median progression-free survival; RFS, recurrence-free survival; mRFS, median recurrence-free survival; CR, complete response; PR, partial response; SD, stable disease; PD, progressive disease.

### 2.2 Differentially expression genes

The R package ‘limma’ (19) is employed to identify differentially expressed genes (DEGs) between poor prognosis (OS<2 years, n = 58) and better prognosis (OS ≥ 2 years, n = 66) in patients based on the mCRC gene expression from the training cohort. with threshold as adjust *P*-value< 0.1 and |log2- fold change|≥1.5.

### 2.3 Functional enrichment analysis

The R packages ‘clusterProfiler’ (20), ‘org.Hs.eg.db’ (21), and ‘stringr’(https://CRAN.R-project.org/package=stringr) were used for Gene set enrichment analyses (GSEA). The ‘clusterProfiler’ package was used to identify and visualize the Gene Ontology (GO) and Kyoto Encyclopedia of Genes and Genomes (KEGG) pathways enriched for DEGs. The Benjamini-Hochberg correction was used to adjust *P*-values for multiple testing, and a q-value threshold of 0.05 was established as the cutoff value.

### 2.4 Establishment and validation of the prognostic model

124 mCRC patients in the GSE72970 cohort were used as a training set to establish the prognostic model. Univariate and multivariate Cox regression analysis was conducted to establish the prognostic model based on the prognostic-related DDR genes using the R package ‘survminer’(https://cran.r-project.org/web/packages/survminer/index.html) and ‘survival’ (22). The following formula was used to calculate the DDRscore, where N is the number of DDR-related gene signature, Exp(DDRgene_i_) is the gene expression value of the DDR-related genes, and Coef(DDRgene_i_) is the multivariate Cox regression coefficient:


$$\text{DDRscore}=\sum_{i=1}^{N}(\text{Coef}\left({\text{DDRgene}}_{\text{i}}\right)*\;\text{Exp}\left({\text{DDRgene}}_{\text{i}}\right))$$

The GSE39582 and TCGA-COAD (stage III/IV) sets were used as validation cohorts to validate the predictive effect of this model. All patients were scored using the uniformed formula and the median score was used to divide the patients into high-DDRscore and low-DDRscore groups. Kaplan–Meier survival analysis and log-rank test were used to evaluate the prognostic relevance of the prognostic model.

### 2.5 Estimation of immune cell Infiltration and immune checkpoint genes

The R package ‘GSVA’ (23) (GSVA: gene set variation analysis for microarray and RNA-Seq data) was employed to quantify the extent of infiltration of 29 immune cell subtypes between high- and low-DDRscore groups. The *P*<0.05 indicated statistical significance. In previous research, Ye et al. analyze the expression of 34 immune checkpoint genes (24). While two genes of ADORA2A and HLA-DRB1 were not found in the mRNA expression profile in our study. Therefore, the expression of 32 immune checkpoints was analyzed.

### 2.6 Prediction of targeted therapeutic sensitivities

The difference in therapeutic sensitivities between high- and low-DDRscore from DDR-related targeted inhibitor therapy was analyzed. Drug sensitivity data of cancer cell lines were obtained from GDSC drug response databases. The half maximal inhibitory concentration (IC50) value of targeted inhibitor was calculated by the R package “oncoPredict” (https://CRAN.R-project.org/package=oncoPredict), including CHK1, ATM, DNA-PK, and PARP inhibitors. Wilcoxon test utilized to assess the IC50 differences between the high-DDRscore and low-DDRscore groups.

### 2.7 Statistical analysis

Univariate and multivariate Cox regressions were conducted by using the ‘survminer’ and ‘survival’ R package. The OS of the high- and low-DDRscore subgroups were compared using the Kaplan–Meier method with log-rank test. Time-dependent receiver operating characteristic (timeROC) were applied to assess the sensitivity and specificity of survival prediction based on the DDRscore, and the ‘survivalROC’(https://CRAN.R-project.org/package=survivalROC) package was utilized to quantify the area under the curve (AUC). The ROC curve was used to assess the prognosis classification performance of the DDRscore and clinical features.The chi-squared test was used to investigate categorical and quantitative data differences between different datasets or groups, respectively. Two tailed *P*<0.05 was used to determine statistical significance. All the statistical analysis and visualization were performed with the R version 4.1.2 (Institute for Statistics and Mathematics, Vienna, Austria 4).

## 3 Results

### 3.1 Identification of differently expressed DDR genes

**Figure 1:**
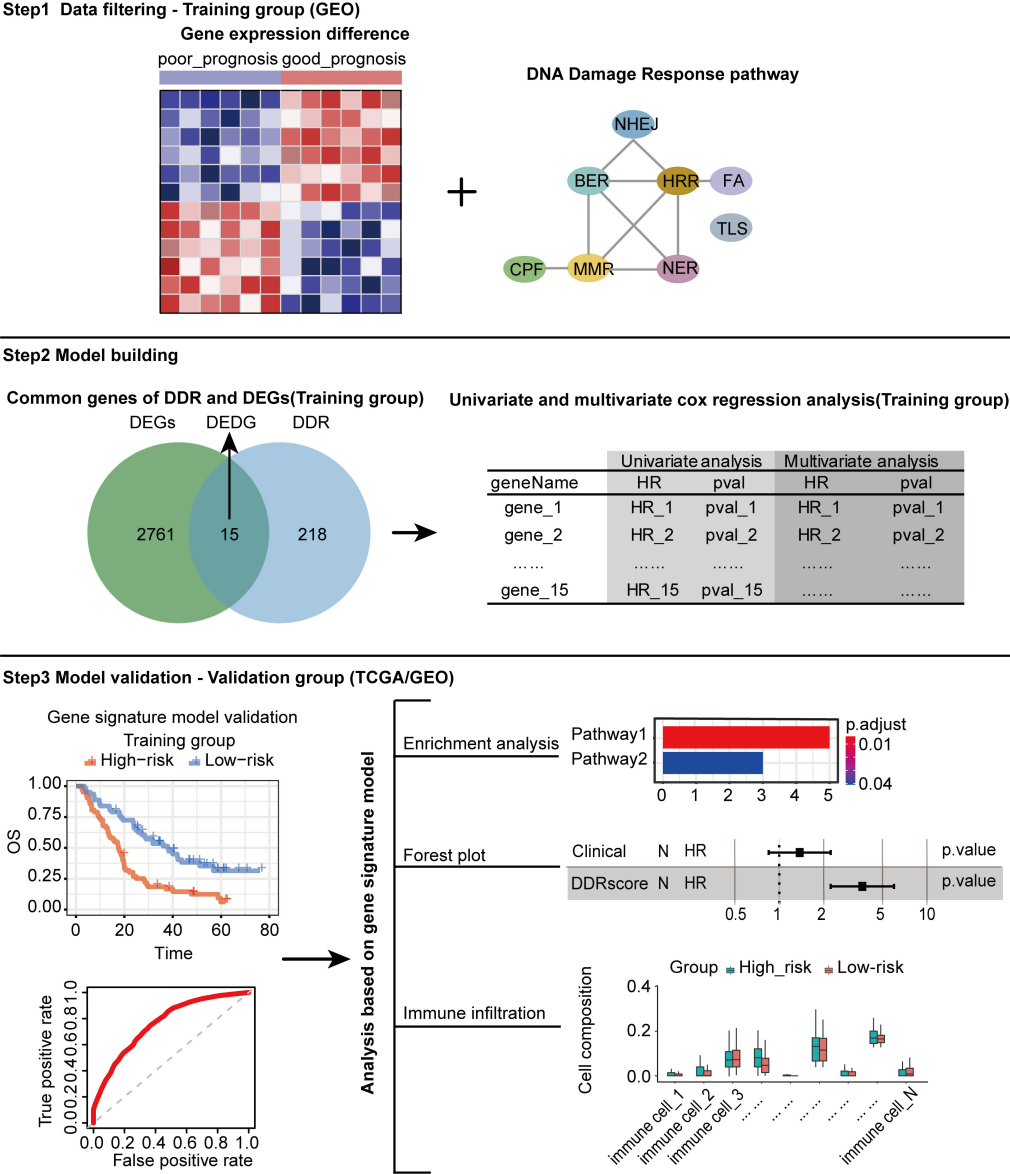
Flow chart of the analytical process in this study.

**Figure 2:**
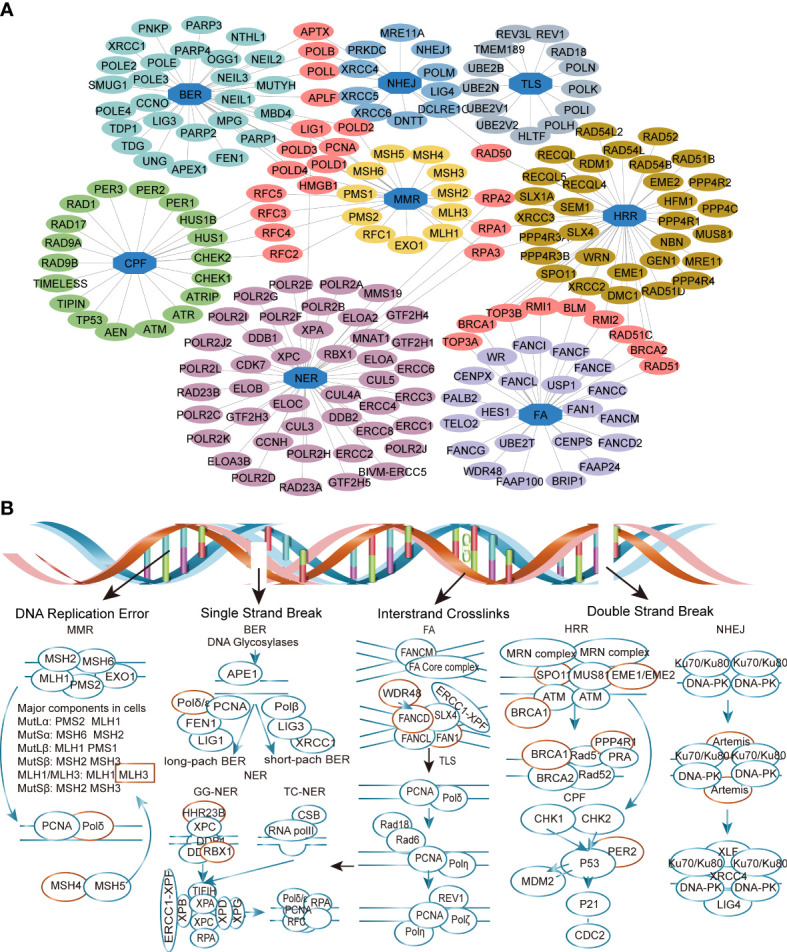
Identification of the differently expressed DDR genes (DEDGs) in metastatic colorectal cancer (mCRC). **(A)** Co-network of DDR genes in different pathways **(B)**The DEDGs distributed in the DDR pathway. The red circle represents DEDGs. MMR, mismatch repair; BER, base excision repair; FA, Fanconi anemia; HRR, homologous recombination repair; NHEJ, nonhomologous end-joining; NER, nucleotide excision repair; TLS, translesion DNA synthesis; CPF, checkpoint factors.

The analysis mentality of this study is shown in **Figure 1**. A total of 666 CRC patients, including three separate cohorts GSE72970, GSE39582, and TCGA-COAD, were enrolled in this study (**Table 1**). DDR genes play a crucial role in CRC tumorigenesis development (25), so we want to investigate whether DDR genes exist in DEGs. As shown in the Volcano plot map, 15 DDR genes (*EME2*, *MLH3*, *FAN1*, *WDR48*, *SPO11*, *RAD23B*, *DCLRE1C*, *PER2*, *EME1*, *RBX1*, *MSH4*, *BRCA1*, *PPP4R1*, *POLD1*, and *FANCD2*) were identified among 2776 DEGs in training cohort (**Figure 2**). GO analysis and KEGG pathway enrichment of DEGs in cluster profiles were performed to investigate the functions of the DEGs. *P*< 0.05 and |logFC| > 0.58 were considered to indicate a statistically significant difference. GO analysis showed the DEGs mainly enriched in DNA recombination, double-strand break repair, reciprocal homologous recombination, and homologous recombination (**Supplementary Figure 1A**). KEGG pathway analysis displayed that the DEGs were majorly enriched in the Fanconi anemia pathway, Homologous recombination, Nucleotide excision repair, and mismatch repair (**Supplementary Figure 1B**).

The samples were well clustered into poor (OS<2 years) and better (OS ≥ 2 years) prognosis groups when the DEGs were selected for unsupervised hierarchical clustering (**Figure 2**). After univariate and multivariate Cox regressions analysis of these 15 differentially expressed DDR genes (DEDGs), four DEDGs (*EME2*, *MSH4*, *MLH3*, and *SPO11*) were identified closely associated with the prognosis of mCRC (**Table 2**). The Cox regression model was then applied to construct a prognostic model for the OS of mCRC patients by using the gene expression data of the four DEDGs in the training cohort.

**Table 2:** Univariate and multivariate Cox regressions analysis of differentially expressed DDR genes.

	Univariate analysis				Multivariate analysis			
geneName	HR	*P*-value	lower.95	upper.95	HR	*P*-value	lower.95	upper.95
EME2	4.208	0	2.12	8.352	3.09	0.003	1.468	6.502
MSH4	1.897	0.001	1.293	2.782	1.717	0.008	1.149	2.568
MLH3	2.093	0.011	1.181	3.71	1.889	0.056	0.985	3.622
SPO11	2.428	0.02	1.147	5.141	2.162	0.1	0.864	5.412
WDR48	0.588	0.051	0.345	1.002	–	–	–	–
EME1	1.819	0.063	0.968	3.42	–	–	–	–
RAD23B	1.65	0.089	0.927	2.937	–	–	–	–
FANCD2	1.906	0.091	0.901	4.028	–	–	–	–
FAN1	1.51	0.19	0.816	2.796	–	–	–	–
BRCA1	1.344	0.194	0.86	2.101	–	–	–	–
PPP4R1	1.283	0.241	0.846	1.947	–	–	–	–
POLD1	1.304	0.268	0.815	2.086	–	–	–	–
DCLRE1C	1.313	0.305	0.78	2.21	–	–	–	–
RBX1	1.243	0.372	0.771	2.003	–	–	–	–
PER2	0.889	0.627	0.553	1.43	–	–	–	–

HR, Hazards ratio.

### 3.2 Construct a four-DDR gene signature of mCRC in the training cohort

**Figure 3:**
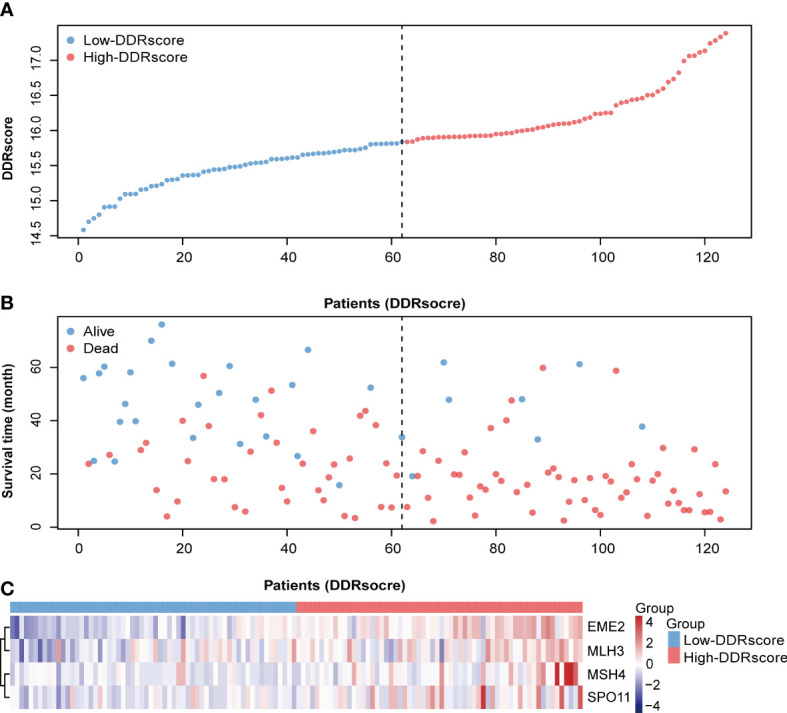
Distribution of metastatic colorectal cancer (mCRC) patients based on the DDRscore. **(A)** Risk curve for the DDRscore of each mCRC case. **(B)** Scatter plot for survival status of each mCRC case, red and blue dots represent death and survival, respectively. **(C)** Heatmap showing the expression profiles of four DDRs in the high- and low-DDRscore groups.

The four DEDGs expression DDRscore (DDRscore = 1.128 × EME2 + 0.541 × MSH4 + 0.636 × MLH3 + 0.771 × SPO11) for each sample was calculated. According to the median cutoff value, the mCRC patients were divided into high- and low-DDRscore groups. A prognostic curve and a scatter plot were used to indicate the DDRscore and the survival status of each mCRC patient (**Figure 3B**). Moreover, most alive cases were mainly distributed in the low-DDRscore group (**Figure 3**). In addition, the heat map of the expression profiles of candidate DEDGs demonstrated that *EME2*, *MSH4*, *MLH3*, and *SPO11* were all highly expressed in the high-DDRscore group (**Figure 3**). In summary, these findings presented the DEDGs as the prognostic signature for mCRC patients.

### 3.3 The prognostic value of four-DEDGs Signature

**Figure 4:**
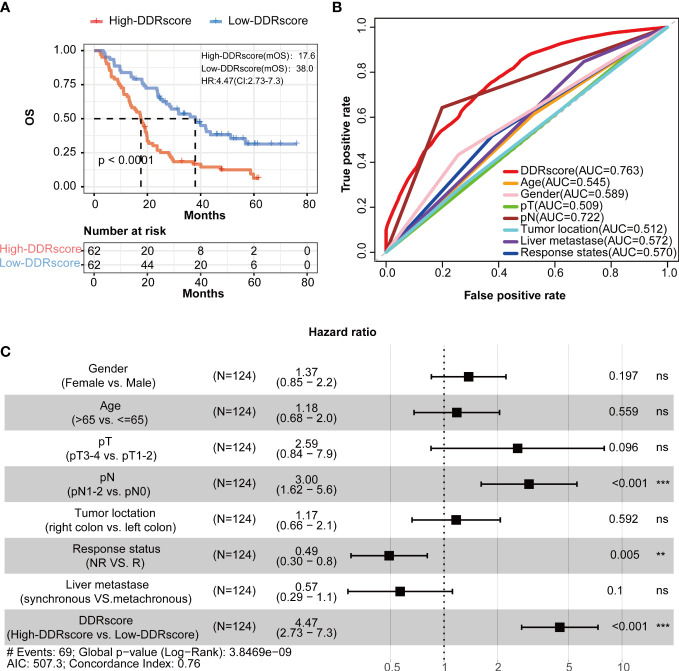
Identification of four-DEDG with prognostic value in metastatic colorectal cancer (mCRC) patients. **(A)** Kaplan–Meier analytical evaluation of the prognostic values of DDRscore. **(B)** Time-dependent receiver operating characteristic curves for the prognostic model based on DDRscore in the training cohort. **(C)** The multivariate Cox regression analysis of DDRscore and clinical features regarding prognostic value. Clinical features: gender, age, pT, pN, and liver metastase, tumor location, and therapy response. * Indicates *P*< 0.05, ** Indicates *P*<0.01, ***Indicates *P*<0.001, ns indicates no significant difference.

Kaplan–Meier analysis presented that mCRC patients have a significantly worse overall survival in the high-DDRscore group than those in low-DDRscore group (**Figure 4**, *P* < 0.001). The time-dependent ROC analyses showed that the AUC of the DDRscore model was 0.763, much higher than other clinical characters’ AUC (0.545 for age, 0.589 for gender, 0.509 for pT stage, 0.722 for pN stage,0.512 for tumor location, 0.572 for live metastate, and 0.570 for the response. states) (**Figure 4**). Univariate Cox analysis displayed that DDRscore (Hazard ratio 4.47, 95% CI 2.73 to 7.3, *P* <0.001) as well as pN (Hazard ratio 3.00, 95% CI 1.62 to 5.60, *P* <0.001) were independent indexes for predicting prognosis of mCRC patients (**Figure 4**). All these data demonstrated this four DEDGs signature’s superior specificity and sensitivity to other clinical parameters.

**Figure 5:**
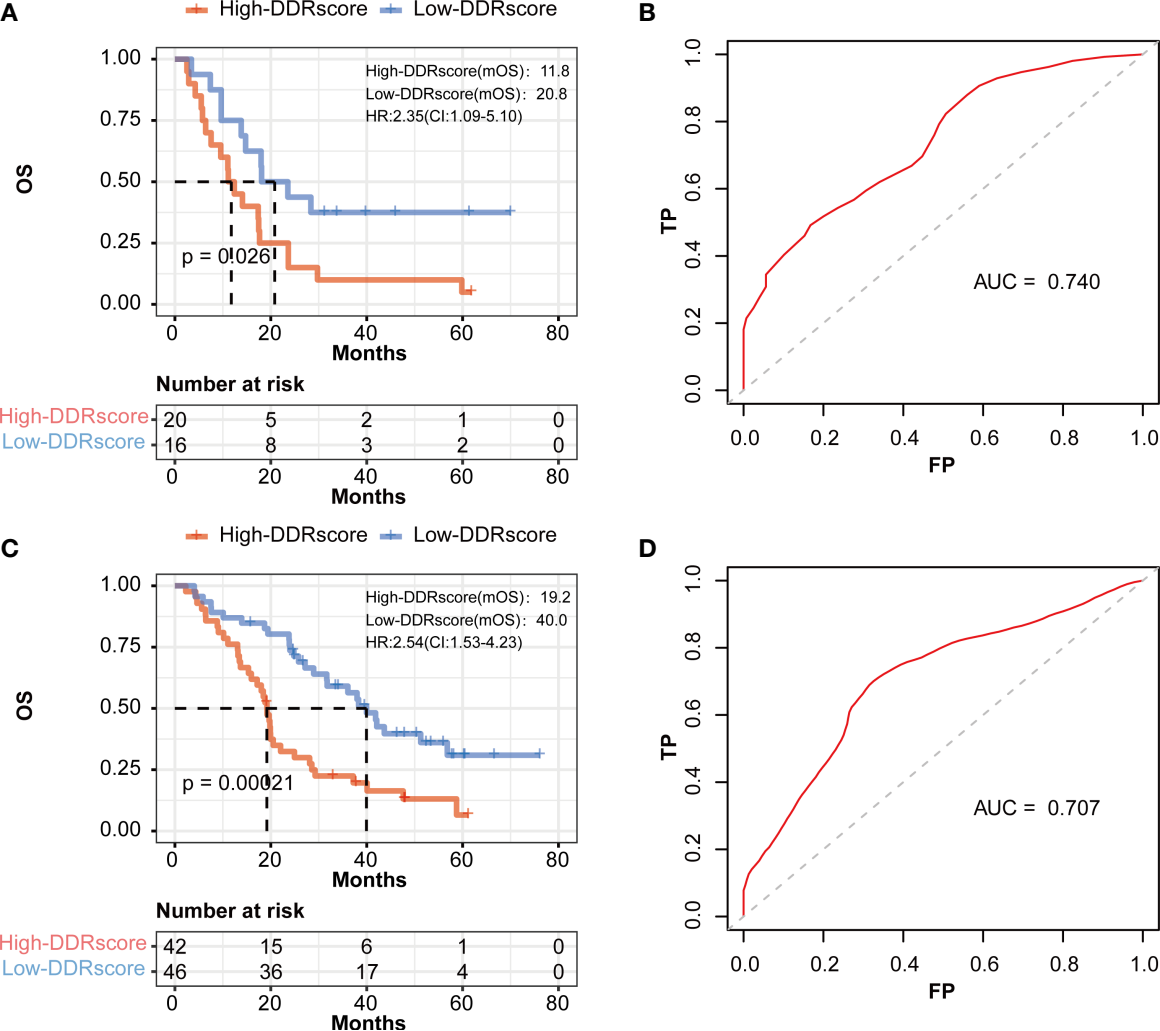
The prognostic value of the DDRscore model in the different sides of colon in mCRC patients. **(A)** Kaplan-Meier plot for OS based on DDRscore of four-DEGE gene signature of patients with LmCRC and RmCRC(B) in the GSE72970-LmCRC cohort. **(B)** AUC of time-dependent ROC curves in the GSE72970-LmCRC cohort. **(C)** Kaplan-Meier plot for OS based on DDRscore of four-DDR gene signature of patients with RmCRC(B) in the GSE72970-RmCRC cohort. **(D)** AUC of time-dependent ROC curves in the GSE72970-RmCRC cohort.

To explore whether different sides of mCRC impact this DDRscore model’ accuracy, we divided the training group into left- and right-side metastatic colorectal cancer (LmCRC, RmCRC) cohorts. Researches show that left- and right-side colorectal cancer are significantly different in clinical features and prognosis (26, 27). The Kaplan–Meier curves show that the survival of LmCRC/RmCRC cohort cases in the high-DDRscore group was significantly lower than those in the low-DDRscore group (**Figures 5A, C**). The AUC of a time-dependent ROC curve of two cohorts for the survival prediction of the DDRscore model were 0.739 and 0.722, respectively (**Figures 5B, D**). Therefore, the impact of the left- or right-side of mCRC is little in our DDRscore model. The DDRscore showed higher predictive prognosis ability in whole mCRC patients (AUC = 0.763).

### 3.4 Identification of the DDRscore model in validation cohorts

**Figure 6:**
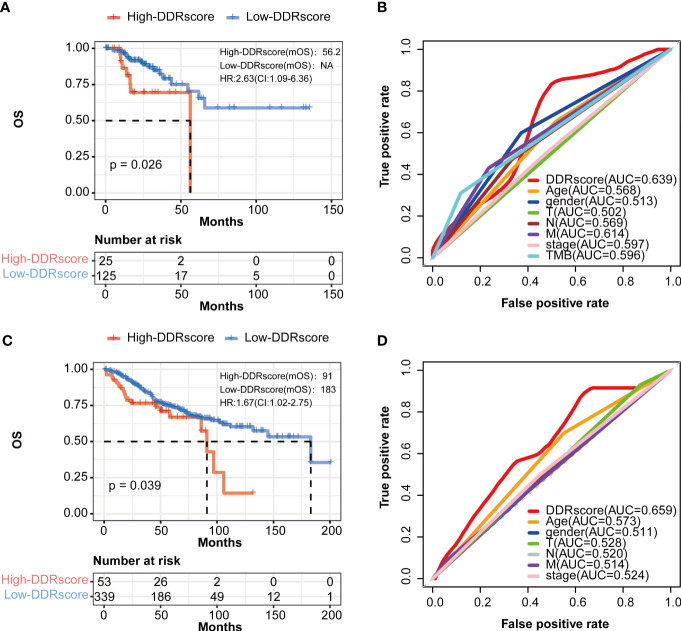
The prognosis of patients with CRC was predicted using the DDRscore in validation datasets. **(A)** Kaplan–Meier analytical evaluation of the prognostic values of DDRscore of mCRC patients from validation dataset of TCGA-COAD. **(B)** Time-dependent receiver operating characteristic curves for the prognostic model based on theDDRscore in the TCGA–COAD (stage III/IV). **(C)** Kaplan–Meier analytical evaluation of the prognostic values of DDRscore of CRC patients from validation dataset of GSE39582. **(D)** Time-dependent receiver operating characteristic curves for the prognostic model based on DDRscore in the GSE39582 cohort.

To demonstrate the DDRscore model’s prognostic generality, we verified this model with a TCGA (N=150, only enrolled stage III-IV patients) dataset containing RNA expression profiling and clinical survival data for CRC patients. Consistent with the results from the mCRC training cohort, the Kaplan–Meier curves of the TCGA cohort revealed that the survival of stage III/IV CRC cases in the high-DDRscore group was significantly lower than those in the low-DDRscore group (**Figure 6**, *P* = 0.026). The AUC of a time-dependent ROC curve for the survival prediction of the DDRscore model was 0.639, also higher than other clinical factors (**Figure 6**). To demonstrate the specificity and sensitivity of this DDRscore model for CRC patients, we further verified this model with a GEO dataset (GSE39582, N = 392). The Kaplan–Meier curves of the GSE39582 cohort revealed that the survival of CRC cases in the high-DDRscore group was significantly lower than those in the low-DDRscore group (**Figure 6**, *P* = 0.039). The AUC of a time-dependent ROC curve for the survival prediction of DDRscore model was 0.659, also better than other clinical factors (**Figure 6**). It demonstrates that this model has good predictive power for the prognosis of mCRC patients and can be extended to the entire CRC patients.

### 3.5 Enrichment and immunity analysis of the DDRscore model

**Figure 7:**
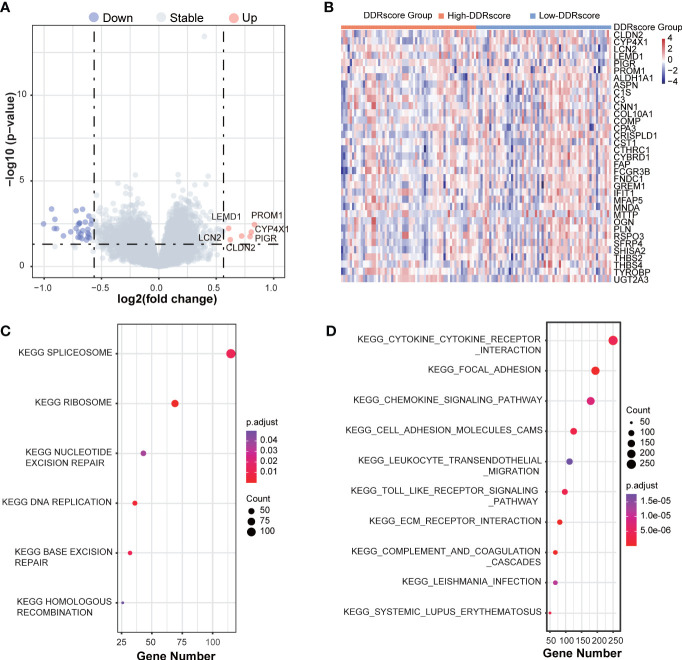
The GSEA function analyses the DEGs between high- and low-DDRscore groups in mCRC patients. **(A)** A volcano plot of DEGs in the high- and low-DDRscore groups. Red indicates upregulated genes, green indicates downregulated genes (high-DDRscore group versus low-DDRscore group), and black indicates no significant difference. **(B)** Heatmap showing the expression profiles of DEGs in high- and low-DDRscore groups. **(C)** The top enriched gene pathways in low-DDRscore group from the discovery cohort applying GSEA algorithm. **(D)** The top enriched gene pathways in high-DDRscore group from the discovery cohort using GSEA algorithm.The color of the ball indicates *P*-value. Blue bars have a less significant *P*-value than red ones.

The DEGs between high- and low-DDRscore groups were screened in the mCRC training dataset, identifying 26 upregulated and 6 downregulated DEGs (**Figure 7**). The heatmap shows the DEGs expression profile of each sample in the high- and low-DDRscore group (**Figure 7**). In the GSE72970 training cohort, GSEA was used to analyze and compare the enrichment of pathways in the high- and low-DDRscore groups. Some DDR-related KEGG pathways, such as nucleotide excision repair, base excision repair, and homologous recombination, were enriched in the high-DDRscore group (**Figure 7**). In contrast, KEGG signaling pathways of cytokine receptor interaction, focal adhesion, chemokine signal pathway, cell adhesion molecules cams, and ECM-receptor interaction were enriched in the low-DDRscore group (**Figure 7**).

**Figure 8:**
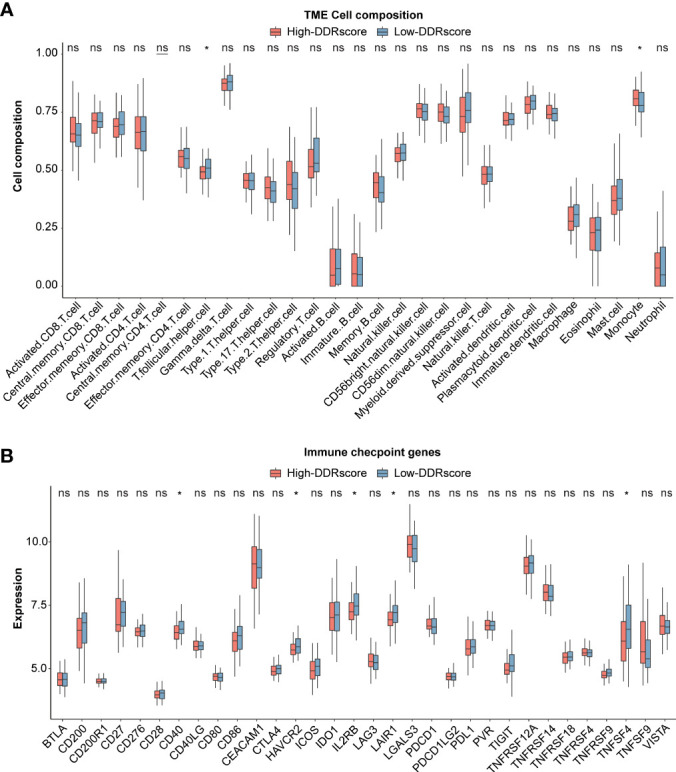
Immune analysis between high- and low-DDRscore groups. **(A)** Boxplot showing the differential abundance of 28 infiltrative immune cells calculated by ssGSEA between high- and low-DDRscore groups in the mCRC. **(B)** Distinct expression of 33 immune checkpoints between high- and low-DDRscore groups in the mCRC. * Indicates P<0.05, ns indicates no significant difference.

ssGSEA revealed the association between DDRscore and immune infiltrating cells. 13 of 29 immune cell types showed higher composition in the high-DDRscore group compared to the low-DDRscore group. Only monocyte and T.follicular.helper.cell were significantly different between high- and low-DDRscore groups in the mCRC cohort (**Figure 8**). Among 32 immune checkpoint genes, CD40, HAVCR2, IL2RB, LAIR1, and TNFSF4 showed significantly increased expression in the low-DDRscore group compared to the high-DDRscore group (**Figure 8**; *P*< 0.05).

### 3.6 Identification of the DDRscore model predictive ability for therapy response

**Figure 9:**
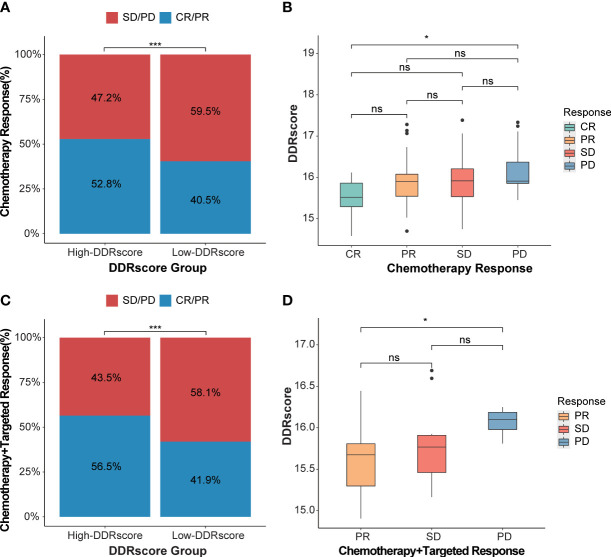
DDRscore model predicts the efficiency of therapy response. **(A)** The proportion of chemotherapy response in high- versus low-DDRscore subgroups. **(B)** Distribution of DDRscore in chemotherapy alone response statuses. **(C)** The proportion of chemotherapy response in high- versus low-DDRscore subgroups. **(D)** Distribution of DDRscore in chemotherapy combined targeted therapy response statuses. CR, complete response; PR, partial response; SD, stable disease; PD, progressive disease. * Indicates P<0.05, ***Indicates P<0.001, ns indicates no significant difference.

To demonstrate our model’s predictive ability for therapy response, we compared the DDRscore value in different response groups. The significant therapeutic response advantages were found in patients with high-DDRscore group compared to those with low-DDRscore group (Chi-square test: chemotherapy alone, **Figure 9**, *P*< 0.001; chemotherapy combined targeted therapy, **Figure 9**, *P*< 0.001). The results showed that the better therapy efficacy, the lower DDRscore value in mCRC patients. In the chemotherapy alone cohort, the DDRscore is highest in the CR group while lowest in the PD group (Kruskal-Wallis, *P*=0.016, **Figure 9**). The same tendency was also identified in the chemotherapy combined targeted therapy cohort, and the DDRscore is significantly lower in the PR group compared with the PD group (Kruskal-Wallis, *P* = 0.023, **Figure 9**). From the above results, it can be concluded that a lower DDRscore may predict better outcomes in this model.

To evaluate the value of DDRscore in predicting the clinical therapeutic efficacy of CRC, we analyzed the difference in sensitivity of DDR-related targeted inhibitors between the groups. Using IC50 value, we calculated the correlation relationship between IC50 of targeted inhibitors and DDRscore. The results showed that CHK1, ATM, and DNA-PK inhibitors displayed higher response sensitivity in cell lines with high DDRscore, especially for ATM inhibitor (KU-55933, *P* = 0.048) and DNA-PK inhibitor (NU7441, *P* = 0.019) (**Supplementary Figure 3D** **)**. While the PARP inhibitors tend to be higher sensitivity with low DDRscore (**Supplementary Figure 3G**).

## 4 Discussion

CRC is a poor prognosis malignancy, especially in mCRC (28). In recent years, many studies explore the diagnostic or prognostic biomarkers in CRC, including genomics DNA (29, 30), mRNA (31), lncRNA (32), metabolic gene (33), circulating tumor DNA (34, 35), methylation (36), and clinical features (37). DDR gene mutations correlated with poor prognosis in breast cancer (BC) patients, and those who harbored the MDC1 gene mutation had the worse prognosis (38). Furthermore, DDR somatic mutations and their co-occurrence are correlated with recurrence-free survival (32). DDR-related ATM or BRCA2 somatic mutations were demonstrated as biomarkers to predict chemotherapy response in stage III CRC patients. Few studies focused on the association between DDR gene expression and CRC prognosis.

An accurate prognostic predictive model may aid physicians in making clinical decisions or guiding adjuvant therapy, especially for vulnerable patients with high mortality risk. The prognostic models based on immune-related genes (39), metabolic genes (36), regulatory factor family genes (40), ferroptosis-related genes (41), m6A methylation regulators (42) and lncRNA (43) have been constructed in CRC. But none of the prognostic models based on DDR-related genes have been established in CRC or other cancers. Herein, we constructed a DDRscore model to predict mCRC prognosis through multivariate Cox analysis based on the four core DDR genes. The GSE72970 cohort was used as a training group. The ROC and survival analysis showed that four DDR genes had excellent diagnostic ability and could distinguish worse prognosis mCRC patients. The validated group of the TCGA cohort revealed similar results to the training group. Moreover, a GSE39582 cohort was selected to verify the model in all CRC patients, which showed consistent results with the training and validated group.The four-DEDGs signature has been proved to have an excellent ability to predict prognosis by Kaplan–Meier analysis, time-dependent receiver operating characteristic (ROC), DDRscore, and univariate and multivariate cox regression analysis based on GEO and TCGA datasets.

In our DDRscore model, *EME2*, *MSH4*, *MLH3*, and *SPO11* high expression predict a worse prognosis in mCRC. EME2 interacts with MUS81 preferentially during the S-phase of the cell cycle, and MUS81-EME2 plays a crucial role in repairing DNA damage and maintaining genomic integrity (44). Furthermore, previous studies reported that EME2 is overexpressed in tumor tissue and was identified as poor prognosis-associated with prostate cancer (45) and gastric cancer (46). MSH4 is a meiosis-specific MutS homolog. MSH4 played an important role during the Cell Cycle, Mitotic, and Meiosis processes (47). MLH3 is a member of the DNA mismatch repair gene family, which is observed with a lower expression level in tumor samples compared with normal tissue. Variants in MLH3 can increase CRC risk (48). SPO11 protein formatted DNA double-strand break (DSB), which initiates recombination and allows chromosome segregation during meiosis (49). Hisham Eldai. et al. identified copy number aberrations (CNAs) of SPO11 in CRCs (50). SPO11 knockdown in various cancer cell lines results in reduced proliferation and altered cell cycle dynamic. *SPO11* may play a critical role in genome stability control and be essential for cancer progression. The SPO11 protein may have diagnostic, prognostic, and therapeutic value in cancer treatment (51). Given all this, these four DDR genes (*EME2*, *MSH4*, *MLH3*, and *SPO11*) have been proved to take part in the pathogenesis, progression, and prognosis of cancers.

Some researchers have demonstrated that mCRCs are clinically heterogeneous. Left- and right-sided mCRC patients have significantly different genomics, prognosis, and clinical characteristics (26, 30). Although left-sided mCRCs show a better prognosis compared to right-sided ones in previous research, our DDRscore model indicates that all enrolled mCRCs (AUC=0.763) are better than those divided into LmCRC9 (AUC=0.722) and RmCRC cohorts (AUC=0.707). Furthermore, the model also exhibits excellent predictive prognosis ability in CRCs (**Figure 6D**, AUC=0.659), not only suitable for mCRCs.

A high tumor mutational burden (TMB), which is linked to greater chances of responding to immunotherapies, is frequently manifested by defects in replication repair-associated DNA polymerases. DDR mutations are linked to an improved OS in CRC patients receiving ICIs (52). In our study, the TMB was higher in the high-DDRscore group compared with the low-DDRscore group **(** **Supplementary Figure 2**, *P* = 0.15). To demonstrate the predicted specificity of DDRscore in the TMB subgroup, we divided the mCRC patients with TMB values into TMB-H and TMB-L cohorts based on the cutoff value of 10 mut/Mb from the TCGA datasets. The prognosis of the low-DDRscore group is better in the TMB-L cohort **(** **Supplementary Figure 2**, *P* = 0.046). The same trend was found in the TMB-H cohort **(** **Supplementary Figure 2**, *P* = 0.089), but not reaching the level of significant difference, possibly account of the too small sample size. The results showed that DDRscore could distinguish the prognosis of patients in the TMB subgroups. To further confirm this finding, a larger cohort must be included due to the included sample’s small size.

The functional enrichment analysis between high- and low-DDRscore groups showed DEGs mainly enriched in nucleotide excision repair, base excision repair, homologous recombination, cytokine receptor interaction, chemokine signal pathway, cell adhesion molecules cams, ECM-receptor interaction, and focal adhesion. These pathways regulate various aspects of the immune response, infection, tumorigenesis, progression, and metastasis in CRC (53–58). High expression of *PROM1*, *LEMD1*, *CLDN2*, *PIGR* and *LCN2* (**Figure 7B**) were associated with CRC cell migration (59, 60), promoting colorectal cancer growth and metastasis (61–63). This could explain the poor prognosis of CRC patients with high DDRscore. Immune checkpoint molecules are essential targets for ICI therapy, and studies suggest that high expression of immune checkpoint molecules is related to superior immunotherapy efficacy (64, 65). In our study, immune cells (such as T.follicular.helper.cell) infiltration and immune checkpoints (CD40, HAVCR2, IL2RB, LAIR1, and TNFSF4) expression were highly enriched in patients with a low-DDRscore group. A better immune microenvironment may explain the preferable prognosis in low-DDRscores patients.

We finally explored the role of DDRscore in predicting the efficacy of different therapies. The primary treatment for unresectable mCRC is systemic therapy (cytotoxic chemotherapy, biologic therapy such as antibodies to cellular growth factors, immunotherapy, and their combinations.) (66). Commonly used chemotherapy regimens, such as FOLFIRI (folinic acid, fluorouracil, and irinotecan), FOLFOX (folinic acid, fluorouracil, and oxaliplatin), and CapeOX (capecitabine plus oxaliplatin), have become the standard of care in advanced CRC (67). But traditional chemotherapy regimens are known to have serious adverse events, such as weaken immuned system, hair loss, constipation dairrhea and neuropathy. These adverse events may be fatal in patients with advanced CRC patients. In the training cohort, the mCRC patients mainly received chemotherapy alone (FOLFIRI or FOLFOX) and chemotherapy combined targeted therapy (FOLFOX+Bevacizumab or FOLFIRI+Bevacizumab). We explored the predictability of DDRscore in two main therapy effects. The results showed that our DDRscore model could evaluate the efficiency of therapy response. The patients with low DDRscore are more likely to benefit from chemotherapy alone or chemotherapy combined targeted therapy. In addition, DDRscore can be used to predict the efficacy of targeted inhibitors. The growing understanding of DNA damage response (DDR) pathways has broadened the therapeutic landscape in oncology over the last few decades. The repertoire of DDR-targeting agents has rapidly expanded to include inhibitors of multiple DDR pathway members, including PARP, ATM, ATR, CHK1, WEE1, and DNA-PK (68). Potential druggable targets and corresponding inhibitors were screened for CRC patients who are defined as high-DDRscore with the constructed prognostic model, and two most promising compounds, namely KU-55933 (ATM inhibitor) and NU7441 (DNA-PK inhibitor), were identified from the GDSC drug response database.

## 5 Conclusion

We constructed and validated a DDR-related model to predict prognosis with mCRC using GEO and TCGA public datasets. The DDRscore was identified as an independent prognosis and therapy response predictor for patients with mCRC. Our results suggested a promising insight into DDR genes, provided a personalized prediction tool for prognosis, and helped to develop new therapeutic targets and prognostic biomarkers in the mCRC population.

## Data availability statement

Publicly available datasets were analyzed in this study. This data can be found here: The datasets of CRC were collected from the Gene Expression Omnibus (GEO) (https://www.ncbi.nlm.nih.gov/geo/) and TCGA-COAD (https://xenabrowser.net/datapages).

## Author contributions

XM and JM: conception and design of study. MW and JS: interpretation of data and drafting the manuscript. JZ: acquisition and analysis of data. SL: revising the manuscript critically for important intellectual content. XM: approval of the version of the manuscript to be published. All authors contributed to the article and approved the submitted version.
