# Supplementary material for: Construction of a DDR-related signature for predicting of prognosis in metastatic colorectal carcinoma
Source: Front Oncol. 2023 Feb 1;13:1043160. doi: 10.3389/fonc.2023.1043160 (PMC9931195; doi:10.3389/fonc.2023.1043160)
Supplement: Supplementary file 1 [file DataSheet_1.docx]

Supplementary Material

## Supplementary Figures


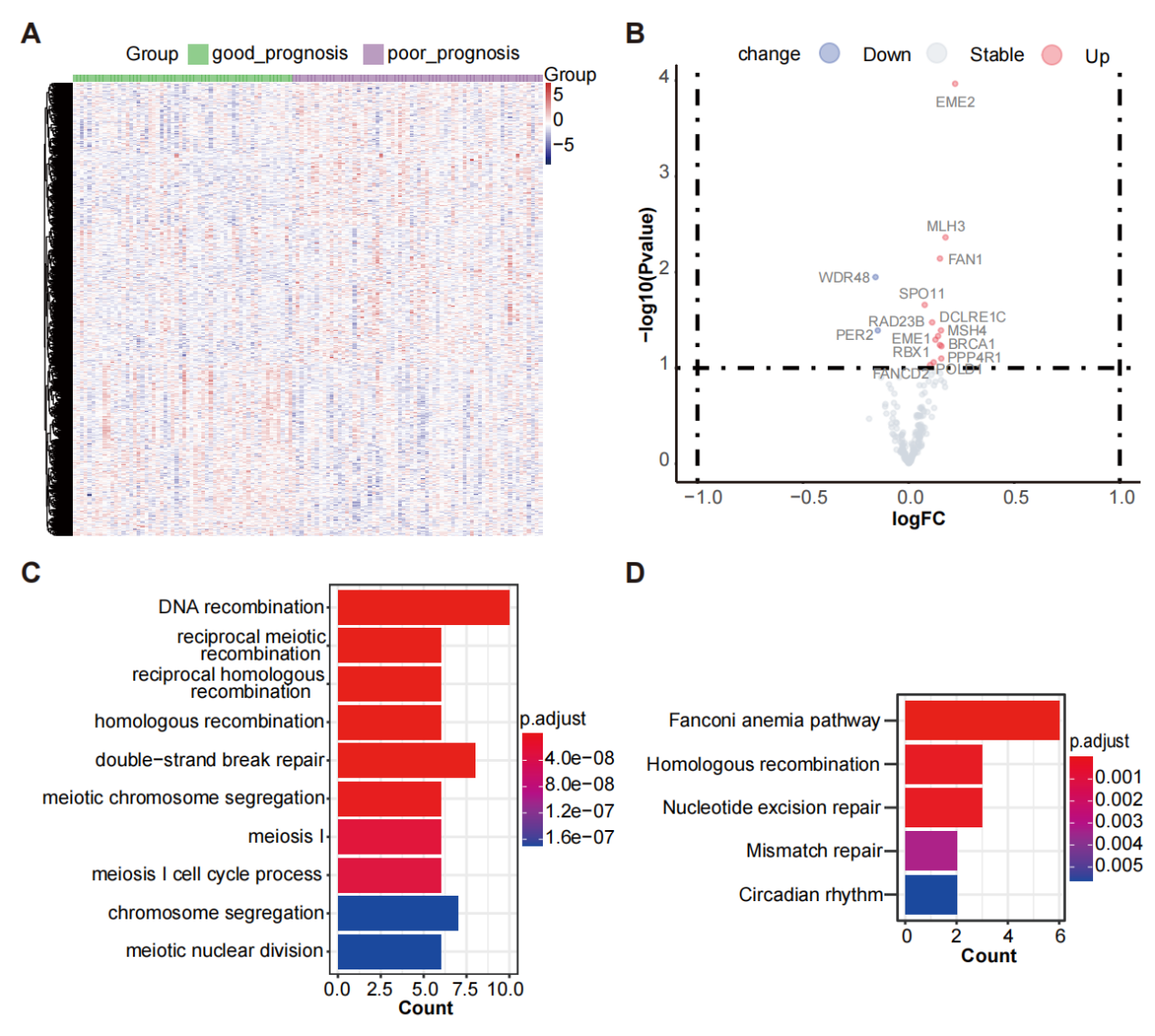


**Supplementary Figure 1**. **The function enrichment analyses of the DDR DEGs.** (**A**) Unsupervised clustering of DEGs heatmap ranked by *P*-value from training cohort. Red: upregulated DEDGs, navy blue: downregulate DEDGs. (**B**) Volcano plot representing the DEGs between the OS＜2 years group and OS＞2 years group of mCRC patients. The upregulated and downregulated DEDGs are highlighted in red and green, respectively. (**C**) GO analysis of the DEDGs. (**D**) KEGG analysis of the DEDGs. DEDGs, differentially expressed DDR genes; DEGs, differentially expressed genes; OS, overall survival.


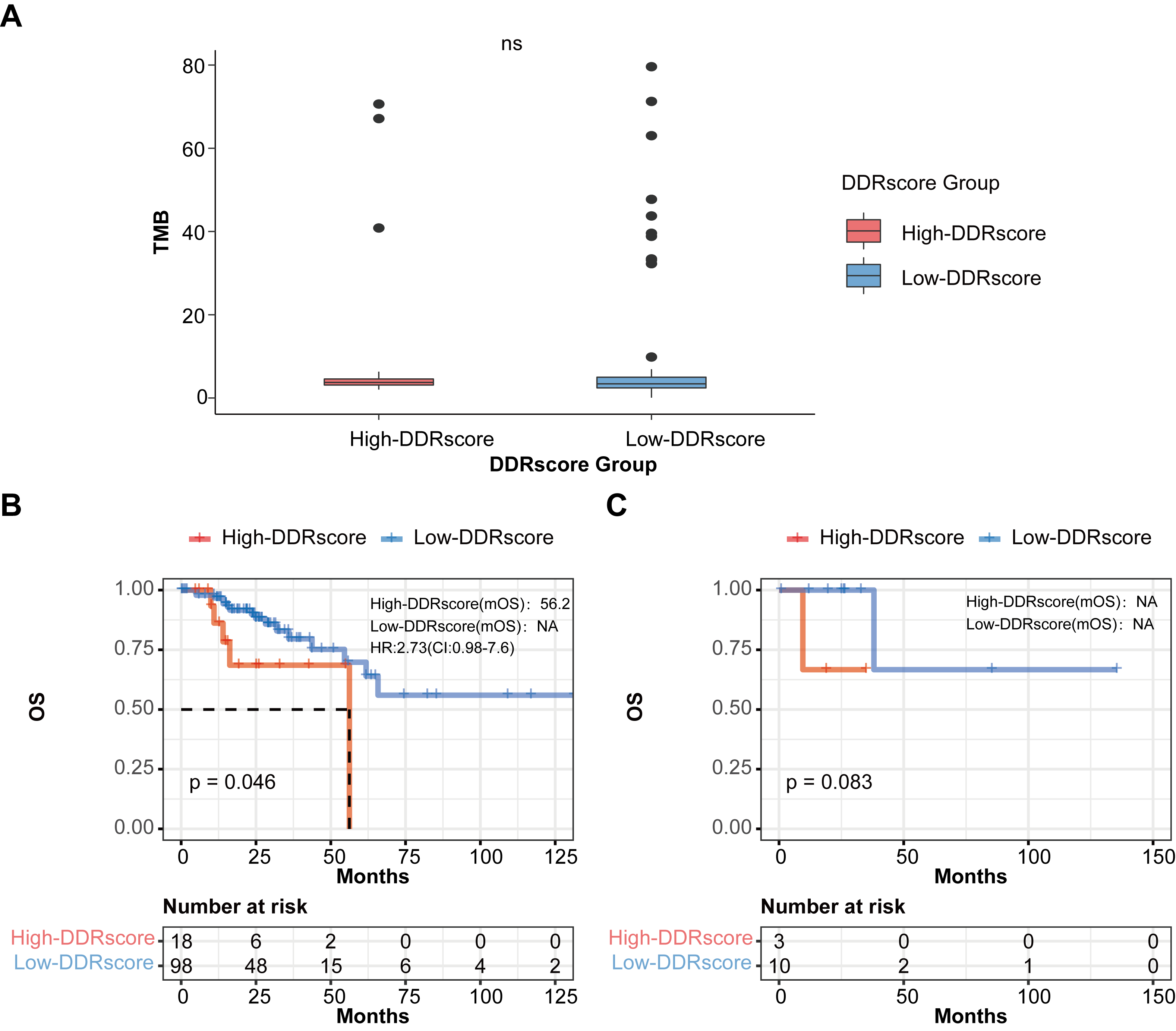


**Supplementary Figure 2. Survival prediction for TMB subtypes using the DDRscore model.** (**A**) TMB value distribution in high- and low-DDRscore groups. (**B**) Survival prediction for TMB-L cohort using the DDRscore model. (**C**). Survival prediction for TMB-H cohort using the DDRscore model.


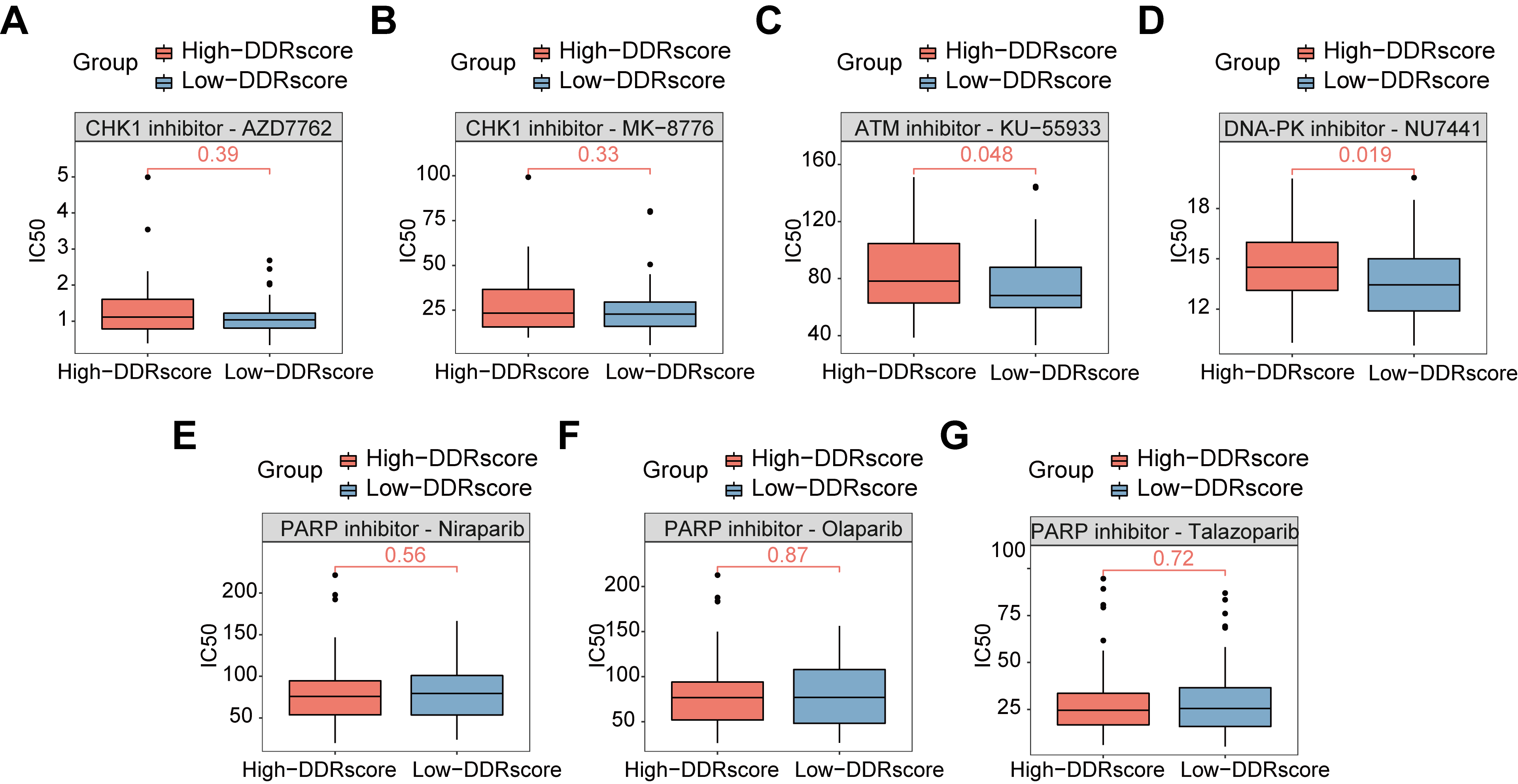


**Supplementary Figure 3. The association between DDRscore and DDR-related targeted inhibitors.** The associations between DDRscore and IC50 value of CHK1 inhibitors of AZD7762 (**A**) and MK-8776 (**B**); ATM inhibitor of KU-55933 (**C**); DNA-PK inhibitor of NU7441(**D**); and PARP inhibitors of Niraparib (**E**), Olaparib (**F**), and Talazoparib (**G**) from the GDSC drug response database.
